# Stretchable Sensors for Soft Robotic Grippers in Edge-Intelligent IoT Applications

**DOI:** 10.3390/s23084039

**Published:** 2023-04-17

**Authors:** Prosenjit Kumar Ghosh, Prabha Sundaravadivel

**Affiliations:** Department of Electrical and Computer Engineering, The University of Texas at Tyler, Tyler, TX 75799, USA; pghosh2@patriots.uttyler.edu

**Keywords:** soft sensor, liquid metal, 3D printing, stretchable electronics, soft grippers

## Abstract

The rapid development of electronic material and sensing technology has enabled research to be conducted on liquid metal-based soft sensors. The application of soft sensors is widespread and has many applications in soft robotics, smart prosthetics, and human-machine interfaces, where these sensors can be integrated for precise and sensitive monitoring. Soft sensors can be easily integrated for soft robotic applications, where traditional sensors are incompatible with robotic applications as these types of sensors show large deformation and very flexible. These liquid-metal-based sensors have been widely used for biomedical, agricultural and underwater applications. In this research, we have designed and fabricated a novel soft sensor that yields microfluidic channel arrays embedded with liquid metal Galinstan alloy. First of all, the article presents different fabrication steps such as 3D modeling, printing, and liquid metal injection. Different sensing performances such as stretchability, linearity, and durability results are measured and characterized. The fabricated soft sensor demonstrated excellent stability and reliability and exhibited promising sensitivity with respect to different pressures and conditions.

## 1. Introduction

With the development of materials science and electronic technology, a variety of new conductive materials are emerging in an endless stream, providing the possibility to prepare functional biomedical electronic devices. There are plenty of applications for soft electronic devices. Soft matter electronic devices have revolutionized the field of wearable computing, biomedical devices, and soft robotics applications. Moreover, every field of application depends on good human-machine interactions that are safe and flexible with human tissue and motion. The sensor is very compatible with the human body because it does not contain any hard material and is capable of complying with natural human tissue that can be used any support natural limb motion. It is crucial to use soft sensors to operate smoothly in health monitoring and soft robotic applications, and traditional sensors are incompatible because they are comprised of electronic circuits and rigid materials. In-room temperature liquid metal-based sensors that are highly flexible and stretchable can be used. This type of liquid metal-based sensor can identify the elastic pressure and shear deformation. Soft matter electronic devices have revolutionized the field of wearable computing, biomedical devices, and soft robotics applications. Moreover, every field of application depends on good human–machine interactions that are safe and flexible with human tissue and motion. The thin film elastomer has been widely focused upon in the field of soft matter engineering. Most of the elastomer sensors are embedded with liquid metal that exhibits a broad range of electronic and sensing functionalities. The sensor is very compatible with the human body because it does not contain any hard material and can comply with natural human tissue that can be used any support natural limb motion. Elastomer-based electronics and sensing devices are very promising though they are still in very nascent stages of development. There are a few challenges such as logic functionality, integration with external hardware, and a proper scalable manufacturing process.

Additionally, due to the availability of flexible and stretchable materials and novel fabrication methods, deformable electrodes are very auspicious for use in stretchable sensors for exciting applications. Flexible sensors have become a substantial attraction in the field of robotics due to their physical properties. Flexible sensors are widely used in soft robotics [1], e-skin [2], and consumer electronics [3]. In particular, soft robotics need a sensor that performs different kinds of tasks in an unstructured environment. Aimed at different kinds of problems, diverse materials have been used to gain high sensitivity and stretchability. However, sensitivity and stretchability are highly dependent on the material’s embedding, depositing, and printing process [4,5]. For microelectromechanical technology, soft materials such as Polydimethylsiloxane (PDMS) and Ecoflex 00-30 are mostly used, as the shore hardness is very low.

Flexible and stretchable sensors have attracted substantial attention because of their characteristics, low modulus, and light weight [6,7]. However, not all of these sensors represent strains over 100%, which is a restriction of large-scale fabrication. At the same time, there are other new features such as delamination and rigid electric materials available in the flexible sensor. Durability is one of the important factors for a flexible sensor; several tests have been conducted after fabrication, and it is necessary to make sure that the sensor is long-lasting enough for real-life application. The variety of Young’s moduli is responsible for the poor durability of rigid conductors and soft materials. To remove the Young’s modulus mismatch, different kinds of materials have been explored. Liquid metal has attracted attention due to its low Young’s modulus and high durability, which is maintained for large strains [8,9]. Moreover, researchers are aware of the physical properties of the liquid material that create stretchable conductors and promising candidates for high-performance stretchable sensors. It is mentionable that Wenlong Cheng fabricated a durable and long-term flexible sensor by using an ionic liquid that brought out the solution of mechanical-mismatch errors [10]. The researcher has invented several types of conductive liquid materials such as eutectic gallium–indium (EGaIn) and Galinstan [11,12,13]. Mercury is also another familiar liquid material, but it is toxic and harmful (Table 1).

Traditionally designed sensors are very rigid, and their surface is not suitable for a body skin or robotic hand. All of these parameters reduce their efficiency and haptic sensing. As a result, it is very important to design and fabricate a tactile sensor with high flexibility and ductility [17,18]. Moreover, in recent years, the design and manufacturing process has made substantial progress in the field of flexible tactile sensors. The main drawback of the tactile sensor is that it can be used only for one kind of sensing application and thus cannot be applicable for a multifunctional haptic feedback system. There is plenty of research going on into achieving more comprehensive tactile sensing. A large number of researchers have already proposed multifunctional tactile sensors with different materials; for instance, Wang et al. [19] introduced the direct writing of laser-induced graphene (LIG) on the textile. The potential application of this sensor is to detect ECG signals.

Silk-nanofiber-based carbon fiber membranes can be used for temperature and sensor sensing [20]. Therefore, it is very significant to develop multifunctional tactile sensors. Different kinds of materials, such as carbon material including carbon nanotubes [21], graphene [22], natural-biomaterial-derived carbon [23], silver nanoparticles [24], hydrogels [25], and liquid metal [26] have been widely used for the fabrication of the tactile sensor. The advantage of liquid metal is that it can be used at room temperature. Liquid metal demonstrates high electrical conductivity and deformability, meaning that it is highly durable and stable for tactile sensing [27]. For sensors, the fabrication process is vital because a slight change of material can drastically decrease the performance of the sensor. For the liquid metal sensor, it is very important to select a suitable fabrication process. There are a few fabrication processes that are commonly used for liquid metal, including mask deposition [28], injecting material into microchannels [29], and direct writing/printing [30]. Most researchers have used their own methods to fabricate sensors; for example, stainless steel has been used for patterning [31], and a rolling brush was used to print on an Eco-flex substrate.

The developed sensor was designed and fabricated for wearable and wireless healthcare applications. Another research group developed a multimode printing process to fabricate flexible electronics [32]. This fabrication process maintains the extrusion printing of elastomer layers and spray printing of liquid metal slurry. Modern designs such as digital light processing (DLP)-based printing have been used for the microfluidic channels for lab-on-chip applications [33]. However, DLP-based printing process is not efficient at all and still need to be improved.

The rest of this research paper is structured as follows. In Section 2, the design and fabrication process of the soft sensor is presented; in Section 3, the potential applications of the soft sensor are presented; in Section 4, different sensing characteristics are measured and compared with current technologies; and in Section 5, future work and conclusions are presented.

## 2. Design and Fabrication Process

Pressure and temperature measuring with static and dynamic interactions between the contact surfaces have been a potential application for artificial skin, robotic arms, and texture recognition in wearable computing. In our prior work related to healthcare applications [34], we explored the use of soft sensors for real-time sensing [35] and integrating edge computing [36] for such applications. In our research work related to integrating haptics in assistive technology, we integrated precise pressure-sensitive gloves for haptic sensing [37]. Elastomer pressure sensors that are very soft, stretchable, and can be adjusted using various methods are very helpful in such sensitive applications. By depositing carbon nanotubes on PDMS, elastomer-based capacitive and strain sensors can detect at least 50 KPa of pressure and accommodate strains up to 150% [38]. Another research group developed pressure sensors by embedding planar circuits that aligned with carbon nanotubes in a polyamide thin film [39]. There has been a lot of attempts to use conductive textiles such as electrical and carbonic paint-treated cotton knit to make a conductive sensor. This kind of textile-based sensor can be used for safe human-machine interaction, and they are very convenient for stretchable applications.

### 2.1. Mathematical Modeling of the Soft Sensor

The total resistance of the flexible sensor depends on the geometry of the sensor. The value of resistivity changes when the sensor is stretched or with changes in any physical parameter. According to resistance law,

(1) $$R=\left[\rho \left(\frac{l}{a}\right)\right]$$

where *R* is the initial resistance of the sensor, ρ represents the electrical resistivity of the liquid metal, *a* is the cross-section area of the sensor, and *l* is the length of the sensor.

**Figure 1:**
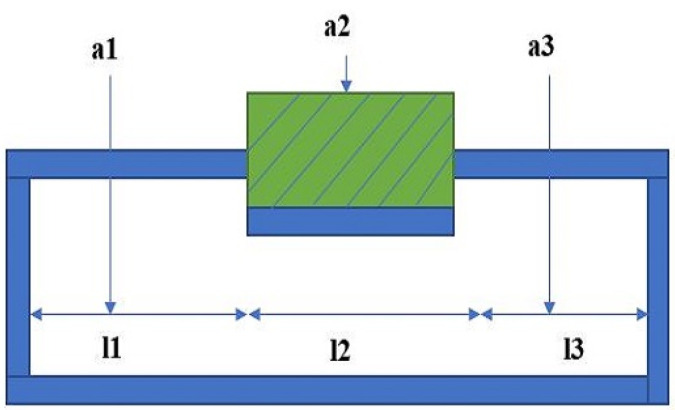
Longitudinal section of the sensor under applied pressure.

Figure 1 shows the ideal conditions of the sensor when the sensor was squeezed under applied pressure. If the sensor is stretched or pressure is applied to the sensor, the physical parameter changes. If the lengths are $l_{1}$, $l_{2}$, and $l_{3}$ and the cross-section areas are $a_{1}$, $a_{2}$, and $a_{3}$, respectively, then the resistance of the sensor can be expressed by

(2) $$\Delta R=\left[\rho \left(\frac{l_{1}}{a_{1}}\right)+\rho \left(\frac{l_{2}}{a_{2}}\right)+\rho \left(\frac{l_{3}}{a_{3}}\right)\right]$$

The resistance change Δ*R* before and after the sensor is extruded can be defined by

(3) $$\Delta R=\left[\rho \left(\frac{l_{1}}{a_{1}}\right)+\rho \left(\frac{l_{2}}{a_{2}}\right)+\rho \left(\frac{l_{3}}{a_{3}}\right)\right]-\left[\rho \left(\frac{L_{1}+L_{3}}{A_{1}+A_{3}}\right)\right]$$

**Figure 2:**
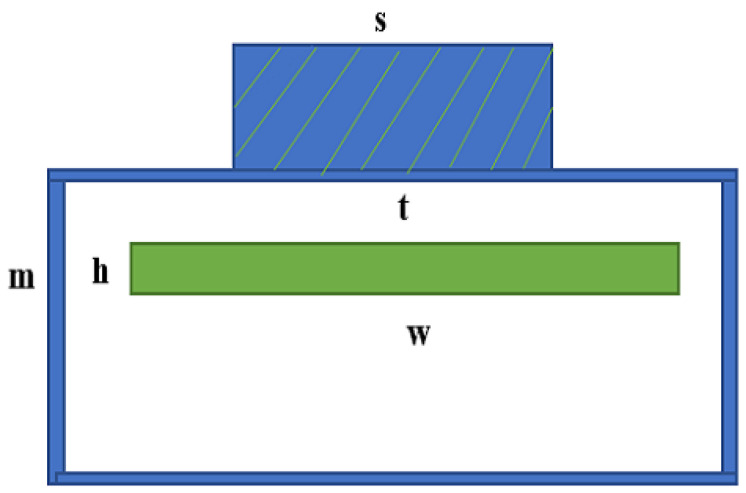
Cross-section view of the soft sensor.

The change of resistance is dependent on the length and the cross-sectional area of the channel. As a result, to measure the sensor resistance, it is very important to consider the length and the cross-sectional area of the sensor properly. In order to get good sensitivity, it is important to investigate the relative size of the sensor from the applied force. To study a good sensor effect, it is important to study the force object and the sensitivity of the sensor. In Figure 2, the lower part of the soft pressure sensor is shown, where w presents the width of the sensor, h represents the height, t is the wall thickness, and m and s are the cylindrical length and width of the sensor. The width of the micro-channel is very narrow with respect to actual production with the application layer.

When the struts are wider than the channel, the pressure from the struts will be distributed around the channel and compress the nearest elastomer. According to Kramer’s study, when the pressure *P* is applied to the upper surface of the elastomer half-space pillar containing the microchannel of conducting liquid, then the relative resistance can be defined as

(4) $$\frac{\Delta R}{R_{0}}=\left[1-{\left(\frac{2wP}{Bh}\right)}^{-1}-1\right]$$

However,

(5) $$R=\left[\rho \left(\frac{w}{h}\right)\right]$$

where *R* is the initial resistance without deformation ρ represents the resistivity of the liquid metal, and *B* is the bending modulus. If the applied pressure is *P*, then the equation of *P* can be described as

(6) $$\Delta R=\left[1-{\left(\frac{2wP}{Bhs}\right)}^{-1}-1\right]$$

The physical structure of the sensor has great influence on its performance. This sensor is mainly two layers: the bottom layer and the top layer. For manufacture, the bottom layer of the sensor is designed using a computer-aided design tool.

### 2.2. Liquid Metal Preparation

Galinstan is a liquid metal that is a combination of gallium, indium, and tin. Their composition ratio is 68.5% Ga, 21.5% In, and 10.0% Sn (by weight). Liquid metal alloys such as Galinstan are often used for different sensor applications as well as some computer hardware applications (Table 2).

**Figure 3:**
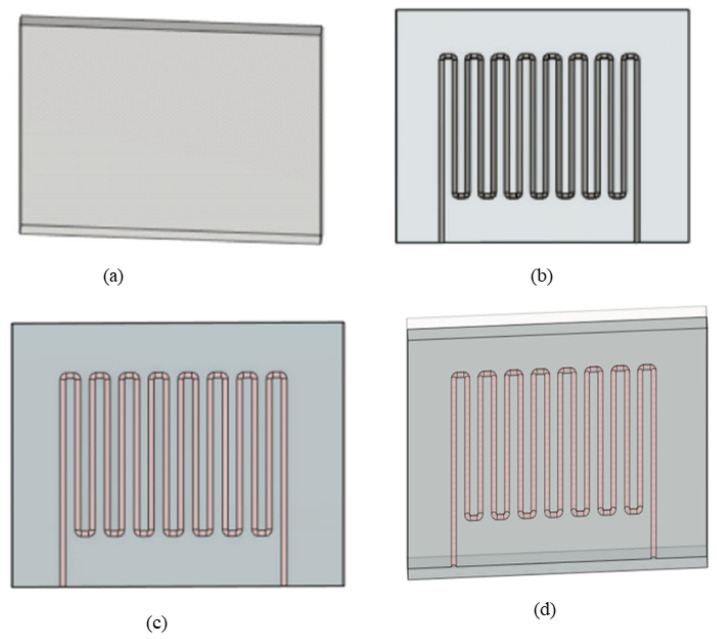
(**a**) Three-dimensional design layout of the soft sensor, (**b**) embedded microchannel, (**c**) schematic view of flexible sensor embedded with liquid metal (injection of Galinstan). (**d**) Schematic illustration of the sensor (Galinstan coating).

**Figure 4:**
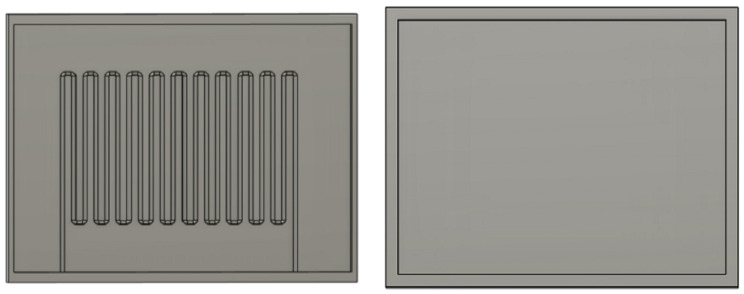
The bottom lid of the sensor (pre-mold processing) and top lid for the fabrication of the soft sensor.

Figure 3 and Figure 4 present the 3D design of the soft sensor. The physical design of the bottom and top layers has been demonstrated, where the top layer is flat and the microchannel is created in the bottom layer. The Fused Deposition Modeling (FDM) printing method using Ultimaker-3 was used for the mold printing of the sensor. As shown in Figure 4, the microchannel is created for the injection of liquid metal. The size of the sensor is 30 mm × 24 mm. The fluidic electrodes are embedded in silicone rubber. The top and bottom sealing layers have been used for the dielectric layer. By using the soft elastomer, the concentration between electrodes changes and gives a wide range of resistivity. For this research, Ecoflex (00-50) has been used, which has a shore hardness of 00-50, tensile strength of 315 psi, and a modulus of 83 KPa. At first, the elastomer Ecoflex is mixed in a 1:1 ratio to fabricate the bottom and top layers. A spin coater (KW-4A) is used for the proper mixing of the elastomer. It was rotated using a glass disk at a speed of 1000 rpm. Moreover, the important factor is to remove the bubble from the mixer. Otherwise, this might affect the performance of the sensor. A degas chamber (5CFM vacuum) is used to clean the air bubbles after which the silicone is filled in both the top and bottom layers. They are cured for around 4 h at 80 °C. The mold and bottom layer are separated carefully so that the microchannel is unaffected.

**Figure 5:**
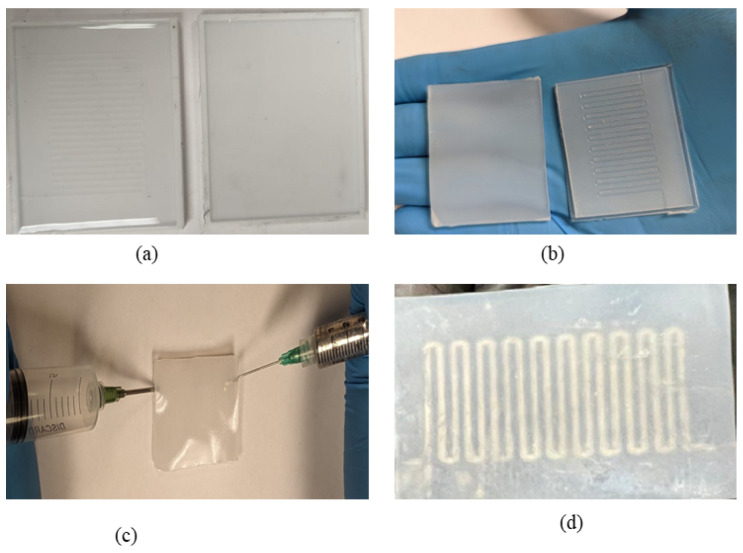
(**a**) Elastomer preparation phase, (**b**) top and bottom layer after curing the elastomer, (**c**) liquid metal injection in the microchannel, (**d**) fabricated sensor after liquid metal injection.

Moreover, liquid metal preparation is very challenging as every metal has a different melting temperature. Galinstan is a liquid metal that is a combination of gallium, indium, and tin. Their composition ratio is 68.5% Ga, 21.5% In, and 10.0% Sn (by weight). Liquid metal alloys such as Galinstan are often used for different sensor applications as well as some computer hardware applications. At first, the metals were taken in proper ratios and placed in a beaker and then heated to 220 °C for almost 1 h. A glass rod was used during the heating process, which helped to mix the metal alloy properly. After melting the metals, they were injected into the micro-channel by using a syringe. For the fabrication process of the soft sensor, a very small amount of Galinstan alloy was used. Finally, the top layer was aligned carefully with the bottom layer. Again, it was kept for curing properly. Figure 5 shows the final fabrication of the sensor.

### 2.3. Device Working Mechanism

This flexible and stretchable sensor operates based on a deformability-dependent resistive sensing mechanism, which can be described by the equation

(7) $$\Delta R=R-R_{0}$$

(8) $$\Delta R=\rho \left[\frac{\left(l+\Delta l\right)}{\left(w+\Delta w\right)}\right]-\rho \left(\frac{l}{wh}\right)$$

The equation shows the apparent resistivity change of the stretchable sensor. Here, *R* is the base resistance of the microchannel when there is no stretch,

$R_{0}$ is the value of resistance when the sensor is stretched,
ρ represents the electrical resistivity of the Galinstan,
*l* is the length of the micro-channel,
Δ*l* is the channel length when the sensor is stretched,
*w* is the width of the sensor, and
*h* is the cross-section of the micro-channel.

**Figure 6:**
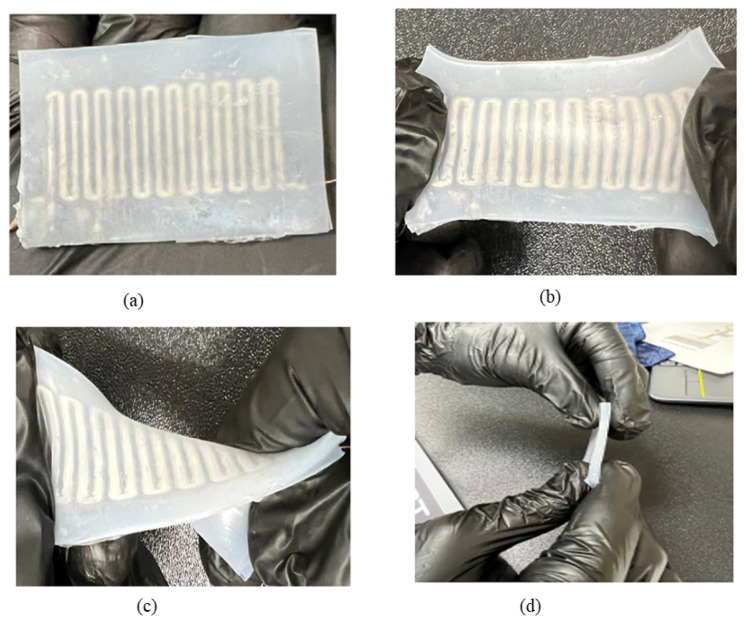
(**a**) The physical diagram of the preparation process of the liquid metal soft sensor. (**b**) Schematic diagram of the preparation process (stretching condition). (**c**) Schematic diagram after fabrication (bending condition). (**d**) Image of the fabricated stretchable sensor (thickness).

The surface of the devices is stretched when the microfluidic channel experiences the applied pressure on it and deformation occurs. The deformation leads to a decrease in the cross-sectional area and helps to increase the channel length. As a result, the value of resistance increases across the microfluidic channel. When the pressure is released, the microfluidic channel recovers soon and goes back to its original state due to the elastic property of the Ecoflex. At the same time, the value of resistance goes to the initial value. In this way, the sensor exhibits an increase and decrease in the electrical resistance of the Galinstan corresponding to the characteristic response of different mechanical forces. In Figure 6, the fabrication process is presented, including elastomer preparation and liquid metal injection.

The liquid metal-based flexible and stretchable sensor shows different types of distinctive features such as superior thinness, flexibility, small size, and comfortability. It isshown that the sensor is effective and robust, and the microchannel sensor should be a high degree of flexibility, conformability, and stretchability. A few physical characteristics such as stretching, bending, and twisting are important for sensor application. In order to measure the performance of the soft sensor, the applied pressure distribution of the Galinstan-based micro-channel sensor corresponding to stretching, bending, and twisting is analyzed. At the stretch conditions, the pressure is distributed uniformly. The sensors show up to 200% stretchability even under high-stress conditions. However, the microchannel shows almost linear behavior concerning applied pressure. That makes the relationship between deformation and resistance change. As a result, the sensor does not experience failures due to high-stress fatigue damage. To evaluate the performance of the sensor, the device is handled meticulously for the loading test, and the output of the microchannel is observed.

## 3. Applications of Soft Sensors

Elastomer-based sensors have a plethora of real-time applications as they exhibit a different type of stretchability and comfortability. Some potential applications of soft sensors are in robotics, artificial skin, health monitoring, and human motion interaction such as breathing, blinks, bending of joints, etc. These types of sensors can be implanted in clothes or directly laminated on human skin, which can produce excellent feedback for health monitoring and other activities. Moreover, a potential application of soft sensors is in the soft robotic field. Soft sensors can be used to measure the direction of a robot, different arm joint angles, the position of objects, etc. These characteristics are applicable to improve the accuracy and control of the robot. Some robotic applications such as grasping and manipulation are crucial when an attached soft sensor can help to achieve these factors. Overall, the use of soft sensors can enhance the controllability, accuracy, and performance of robots. However, there are still some challenges for the wide application of soft sensors. Most soft sensors can deform in only one direction and perform one job. In addition, these sensors have high modulus materials and weak adhesion structures that might be the reason for the lack of comfort of the users. Additionally, integration with other devices such as Bluetooth, thin-film batteries, and packaging is a concerning issue for the soft sensor.

## 4. Results and Discussion

The sensor demonstrates prominent mechanical performance that does not represent any structural damage in conditions of high strength. To obtain the output from the sensor, different apparatuses have been used for this study. To apply fixed pressure on the sensor, a pressure gauge was used, and for storing and analyzing data, KEYSIGHT E4089A, Arduino MKR-1000, and MATLAB were used. The flexible Galinstan micro-channel sensor was designed to accomplish mechanical changes, although the minimum requirement was not only high mechanical durability but also high sensitivity. The sensor working mechanism was the resistivity change with respect to the cross-sectional area of the micro-channel when the sensor was stretched. The value of resistance became larger as the area of the micro-channel became smaller. It was a priority to execute the design function to increase the resistance value and show an outstanding linear relationship with strain.

### 4.1. Stretchability

**Figure 7:**
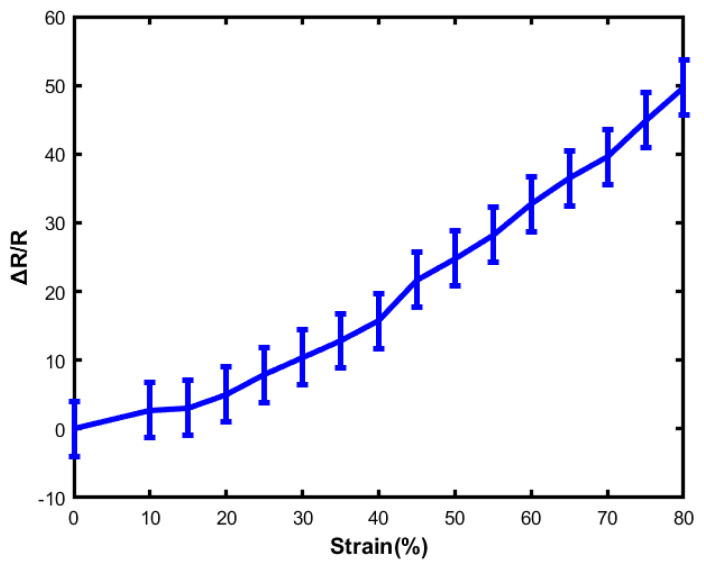
The relationship between strain and resistance change (pressure: 30 kPa).

Stretchability is one of the primary characteristics of flexible sensors. However, most of the traditional sensors are very rigid and brittle. There might be structural damage and performance failure due to large deformations. Elastomer-based sensors show a high stretchability that relies on the physical properties of the microstructure and the component materials. Elastomer-based sensors always present excellent stretchability. Figure 7 represents the relationship between relative resistance change and strain as.

(9) $$\;\mathrm{Strain}\;=\frac{\left(L-L_{0}\right)}{L}=\frac{\Delta L}{L}$$

where *L* is the length of the sensor before being stretched, and $L_{0}$ is the value when the sensor is stretched. Δ*L* represents the value of length change in two different environments. The value of strain is plotted on the X-axis, and the value of relative resistance change is plotted on the Y-axis. Typically, the PDMS/Ecoflex/Elastosil-based sensor’s stretchability depends on the physical properties of the material. Sometimes, their strain range is 0–10% because of the fabrication and lack of strong percolating substrate with a minimum aspect ratio. To fabricate this sensor, an Ecoflex (00-50) was used, which helped the sensor to become very flexible. Though some research groups used carbon nanotube (CNT) films with a highly homogeneous structure, the result showed microcrack propagation and lateral interconnection. The strain of this sensor can be achieved up to 280%, which is applicable for a wide range of applications. However, in this research work, the sensor shows outstanding strain up to 100% and excellent sensitivity. From Figure 7, the relative resistance change is almost linear, and thus the sensor is a potential candidate for different sensing applications.

### 4.2. Gauge Factors

The gauge factor is one of the important indexes for sensitivity. The gauge factor is defined as

(10) $$\;\mathrm{Gauge}\;\mathrm{Factor}\;=\frac{\Delta R/R}{\varepsilon }$$

**Figure 8:**
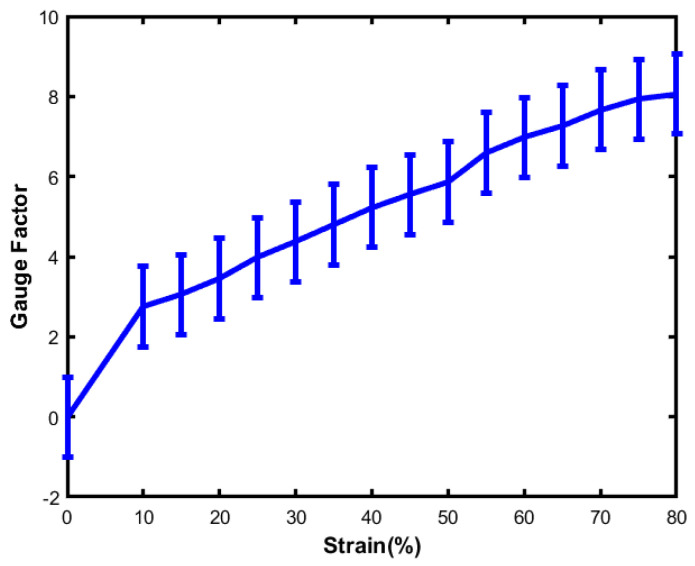
The relationship between the gauge factor and strain.

The gauge factor reflects the sensitivity of the flexible sensor, and the slope of the relative resistance change is defined as the gauge factor. The sensor detects very small deformations when the sensor shows a greater gauge factor. Elastomer/silicone-based liquid metal flexible sensors exhibit a gauge factor with a range of 2–20. Moreover, the gauge factor is controlled by the geometry deformation of the liquid metal. Comparing liquid metals, AgNW/PDMS-based sensors have smaller gauge factors of 2–10, and graphene oxide/PDMS-based strain sensors exhibit a high gauge factor range of 130–260. According to the graph, the value of the gauge factor reaches around 8.06 when the sensor is stretched to about 80%. Both sensors behave identically, although there is a slight change of value with a different strain. The linear characteristics indicate that they are promising candidates for sensor applications. From Figure 8, it is observable that the sensor demonstrated excellent mechanical deformability and did not show any structural damage during mechanical strength changes.

### 4.3. Linearity

Linearity is one of the vital parameters for sensor applications because linearity makes the sensor’s signal accurate and is helpful in observing the output signal. In contrast, nonlinearity represents a deviation from the output of the signal and the measurement of the parameter. As a result, it leads to a complicated calibration process and signal distortion, which indicates poor performance in terms of sensitivity. Galinstan/Ecoflex-embedded based sensors typically exhibit linearity and stretchability. Moreover, the cycling test helps to moderate the linearity by stretching/releasing.

**Figure 9:**
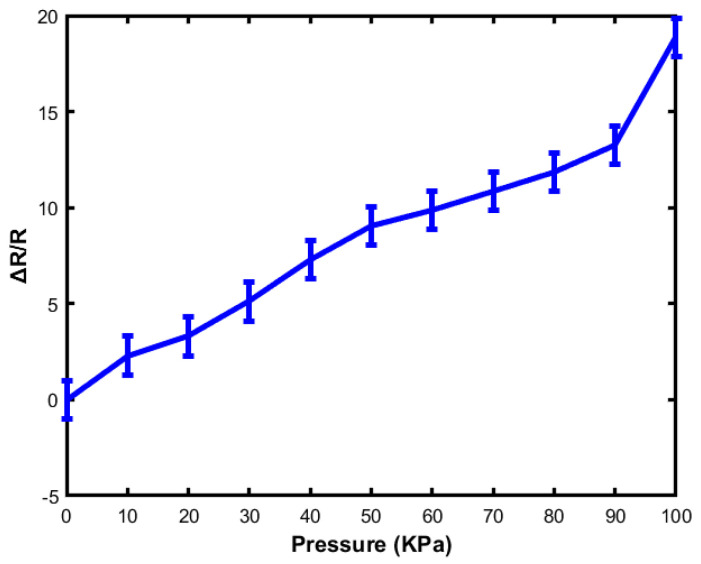
The relative change of resistance with respect to applied pressure.

Figure 9 represents the relative change of resistance with respect to applied pressure on the soft sensor. The range of applied pressure is 10 kPa to 100 kPa with an increment of 10 kPa. From the graph, it is observed that when the applied pressure is 10 Kpa, the relative resistance change is 1.00. However, with the increase in the loading force, the value of resistance changes linearly. Under the same applied pressure, both sensors behave identically. At 50 kPa applied pressure, the value of relative resistance is 1.81 and 1.18, respectively, whereas at 100 kPa applied pressure, the value of relative resistance is 2.97 and 3.14, respectively. Thus, the results indicate that both sensors provide good dynamic response and stability under different applied pressures. Typically, the highly sensitive sensor shows strain with nonlinearity and very low stretchability and represents non-uniform deformation. As a result, the design and fabrication of highly linear, stretchable, and sensitive sensors are still a big challenge. In this research, the fabricated sensor can be used in a wide range of applications as it shows moderate linearity and good response with respect to applied pressure.

Moreover, it is crucial to check the liquid metal condition after stretching. The physical condition of the sensor might be affected, which restricts the sensitivity performance. To measure the conductivity of the sensor, the resistance change is measured after every 150th stretching. It is found that the relative resistance change is 3–7%, which is negligible with respect to elastomer and liquid metal physical conditions. The sensor shows high durability and stability under stretched conditions.

### 4.4. Durability

**Figure 10:**
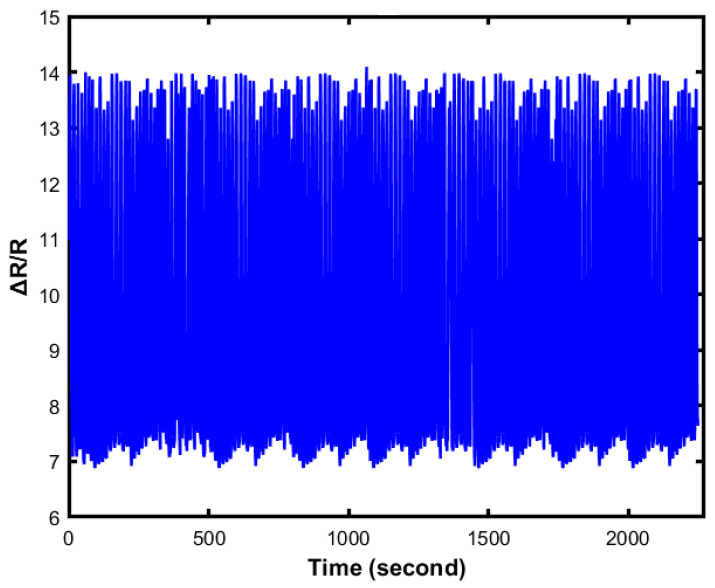
One thousand episodes of cyclic loading and unloading tests at 30 kPa for 6 min.

**Figure 11:**
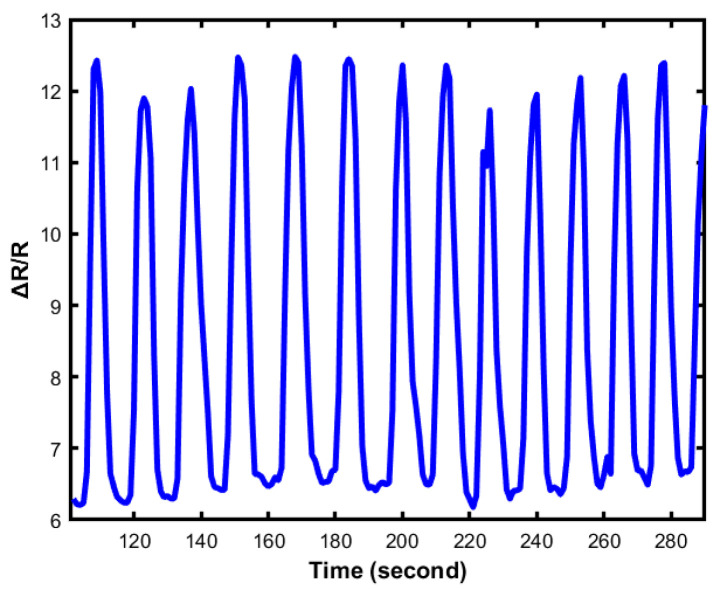
One thousand episodes of cyclic loading and unloading tests at 30 kPa (300 s period).

Durability is vital for flexible devices as the application of the sensor is large and takes extreme dynamic strains (Figure 10 and Figure 11).

The Galinstan/Ecoflex-based sensor shows long-term reliability during the cycling test with a very negligible change in the resistance profile, as shown in Figure 9. The two-layered flexible sensor shows outstanding dynamic bending stability with an almost constant resistance value where the maximum bending angle was used at 90 degrees. More importantly, the lifetime of the sensor highly depends on the sensor’s fabrication. The sensors exhibit high durability that responds to different cyclic loadings with remarkable stability even after thousands of cycles of strain ranging from 10–60%. Figure 10 presents 1000 episodes of cyclic loading and unloading tests when the applied force is 30 kPa. The output of the loading test is generated and plotted. From the figure, it can be observed that the value of resistance changes repeatedly when the cyclic loading and unloading test occurs. Figure 11 shows an enlarged view of the cyclic test. Thus, the sensor demonstrates good durability and repeatability for long-term application.

**Figure 12:**
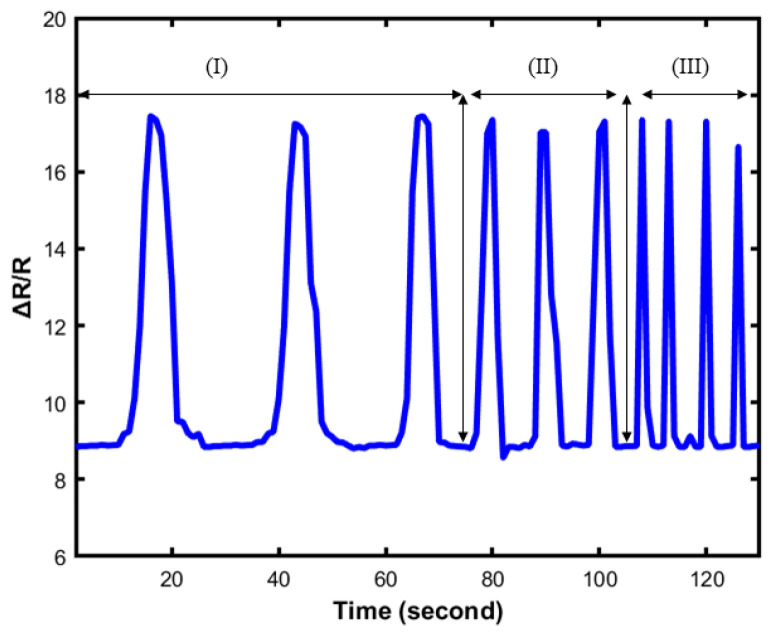
Resistance change under 3 different loading frequencies at 0.020 Hz, 0.08 Hz, and 0.012 Hz marked as region I, II and III respectively.

**Figure 13:**
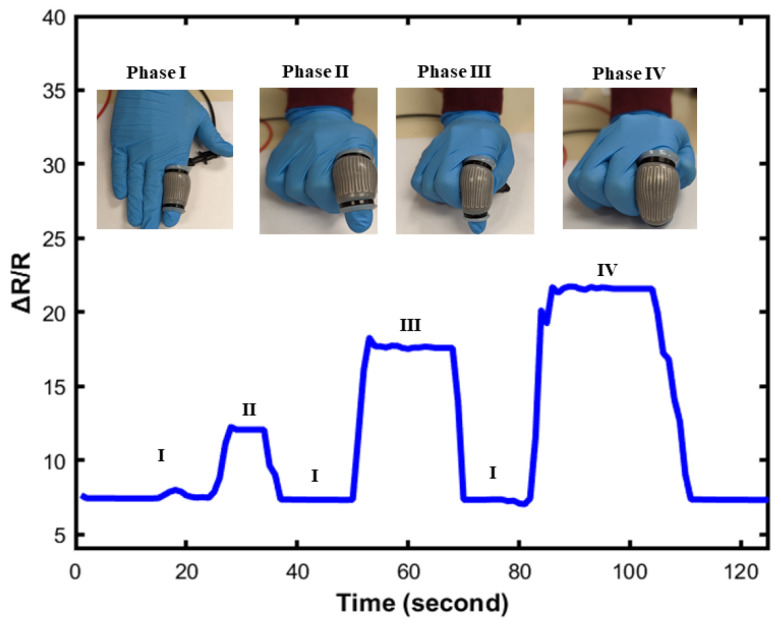
Performance test of the soft sensor using finger positioning.

In Figure 12, the relative resistance change is observed under different applied frequencies. The loading frequencies are 0.020 Hz, 0.08 Hz, and 0.012 Hz. As shown in Figure 12, the value of resistance was almost unchanged under applied frequencies, which indicates that the sensor is stable under different loading frequencies. To further demonstrate the reliability and practicability of the soft sensor, the sensor was attached to a human hand to study different human gestures. Figure 13 presents the proof of concept of its application, mainly focused on dynamic responses. Figure 13 shows the change of the resistance value when the sensor is stretched. The result proves that this soft sensor shows outstanding strain performance and easily recognizes the gesture of the hand (Table 3).

## 5. Conclusions

In this research paper, a new soft flexible sensor has been introduced. The design, fabrication process, performance analysis, and some potential applications are discussed. There are several applications of soft sensors, such as in e-skin, robotics, human-machine interfaces, health monitoring, sports performance analysis, etc. Moreover, it can be predicted that wearable soft sensors will play a vital role in our everyday life, especially in health monitoring devices. However, wearable soft sensors in real-life applications still face some challenges. These types of sensors can detect deformations in one direction, and multi-direction deformation is still a field of research although few strain and pressure sensors have been fabricated and reported. Furthermore, there are a few other obstacles such as high modulation materials and weak adhesion that make the soft sensor not applicable in real life. Overall, this kind of liquid metal sensor shows high performance in terms of stretchability and stability for real-time applications. This fabricated sensor can be used in different forcing conditions. The proposed liquid metal sensor can be implanted as a kind of wearable electronic and used for robotics manipulation applications.

## Figures and Tables

**Table 1:** Comparison of the different liquid metals alloys for sensor fabrication.

Material Name	Electrical Conductivity (S·m${}^{-1}$)	Thermal Conductivity (W·m${}^{-1}$·K${}^{-1}$)	Viscosity (Pa·s)
Mercury [14]	1.06 × 10${}^{6}$	8.54	1.5 × 10${}^{-3}$
EGaIn [15]	3.04 × 10${}^{6}$	26.6	1.9 × 10${}^{-3}$
Galinstan [16]	3.04 × 10${}^{6}$	16.5	2 × 10${}^{-3}$

**Table 2:** Thermal properties of Galinstan.

Properties	Galinstan
Melting point/°C	−19
Boiling point/°C	>1300
Vapor pressure/mmHg	<10${}^{-8}$ at 500 °C
Specific Heat/kJ·kg${}^{-1}$ °C${}^{-1}$	0.200
Density/kgm${}^{-3}$	6440
Thermal	16.5
Surface tension/N·m${}^{-1}$	0.718 at 25 °C
Solubility in water	Insoluble
Viscosity/Pa·s	2.4 × 10${}^{-3}$ at 20 °C

**Table 3:** Fabrication and printing methodology of the sensor.

Materials	Methods	Stretching (%)	GF	Linearity	Applications
EGaIn-PDMS [39]	Printing technology	350	1.6-3.2	partial	Wearable devices
AgNW-PDMS [40]	Micro-modeling	70	2–14	Partly linear	Motion detection
CNT-CB-PDMS [41]	Micromolding method	22.6	29	Nonlinear	Wound monitoring
Graphene-PDMS [42]	Coating techniques	7.1	2.4–14	linear	Wearable devices
GWFs-PDMS [43]	Coating techniques	30	106	Nonlinear	In vitro diagnostic
MWCNTs-PDMS [44]	Liquid phase	45	1.2	Nonlinear	Pressure measurement
AgNP-PDMS [45]	Printing technology	20	4.7–12.5	Two linear	Motions
CNT-PDMS [46]	Printing technology	100	104	Nonlinear	Bending angle
GO-PDMS [47]	Filtration method	(5–2.5 cm)	NA	Nonlinear	Human interactive
Graphene-PDMS [48]	Filtration method	100	7.1	Partly linear	Drug-induced changes
Ti/Au-PDMS [49]	Micromolding method	NA	NA	Nonlinear	Electronic skin
GnPs-PDMS [50]	Micromolding method	10	27.7–164.5	Nonlinear	Human–machine interface
This work	Micromolding method	175	8.6	Partly linear	Robotics, health monitoring

## Data Availability

Not Applicable.
